# Colorectal cancer incidence in *path_MLH1* carriers subjected to different follow-up protocols: a Prospective Lynch Syndrome Database report

**DOI:** 10.1186/s13053-017-0078-5

**Published:** 2017-10-10

**Authors:** Toni Seppälä, Kirsi Pylvänäinen, Dafydd Gareth Evans, Heikki Järvinen, Laura Renkonen-Sinisalo, Inge Bernstein, Elke Holinski-Feder, Paola Sala, Annika Lindblom, Finlay Macrae, Ignacio Blanco, Rolf Sijmons, Jacqueline Jeffries, Hans Vasen, John Burn, Sigve Nakken, Eivind Hovig, Einar Andreas Rødland, Kukatharmini Tharmaratnam, Wouter H. de Vos tot Nederveen Cappel, James Hill, Juul Wijnen, Mark Jenkins, Maurizio Genuardi, Kate Green, Fiona Lalloo, Lone Sunde, Miriam Mints, Lucio Bertario, Marta Pineda, Matilde Navarro, Monika Morak, Ian M. Frayling, John-Paul Plazzer, Julian R. Sampson, Gabriel Capella, Gabriela Möslein, Jukka-Pekka Mecklin, Pål Møller, in collaboration with The Mallorca Group

**Affiliations:** 1 Department of Surgery, Helsinki University Hospital and University of Helsinki, Post Box 340, 00029 Helsinki, Finland; 2 Finnish Lynch Syndrome registry, Helsinki University Hospital, Helsinki, Finland; 3 Department of Education and Science, Central Finland Health Care District, Jyväskylä, Finland; 4 Manchester Centre for Genomic Medicine, Central Manchester University Hospitals NHS Foundation Trust, Manchester, UK; 5 Manchester Centre for Genomic Medicine, Institute of Human Development, MAHSC, University of Manchester, Manchester, UK; 6 Genome-Scale Biology Research Program, University of Helsinki, Helsinki, Finland; 7 Danish HNPCC Register, Hvidovre University Hospital, Copenhagen, Denmark; 8 Department Surgical Gastroenterology, Aalborg University Hospital, Aalborg, Denmark; 9 Medizinische Klinik und Poliklinik IV, Campus Innenstadt, Klinikum der Universität München, Ziemssenstr. 1, 80336 Munich, Germany; 10 MGZ – Medizinisch Genetisches Zentrum, Bayerstr. 3-5, 80335 Munich, Germany; 11 Unit of Hereditary Digestive Tract Tumors IRCCS Istituto Nazionale Tumori Milan, Milan, Italy; 12 Department of Molecular Medicine and Surgery, Karolinska Institutet, Stockholm, Sweden; 13 Colorectal Medicine and Genetics, The Royal Melbourne Hospital, Melbourne, Australia; 14 Department of Medicine, Melbourne University, Melbourne, Australia; 15 Hereditary Cancer Program. Institut Català d’Oncologia-IDIBELL, L’Hospitalet de Llobregat, Barcelona, Spain; 16 Department of Genetics, University of Groningen, University Medical Center Groningen, Groningen, the Netherlands; 17 Institute of Medical Genetics, Cardiff University School of Medicine, Heath Park, Cardiff, CF14 4XN UK; 18 Department of Gastroenterology and Hepatology, Leiden University Medical Centre, Leiden, the Netherlands; 19 Institute of Human Genetics, Newcastle upon Tyne, UK; 20 Department of Tumor Biology, Institute of Cancer Research, The Norwegian Radium Hospital, part of Oslo University Hospital, Oslo, Norway; 21 Institute of Cancer Genetics and Informatics, The Norwegian Radium Hospital, part of Oslo University Hospital, Oslo, Norway; 22 Department of Informatics, University of Oslo, Oslo, Norway; 23 Department of Mathematics, University of Oslo, Blindern, Oslo, Norway; 24 Department of Gastroenterology and Hepatology, Isala Clinics, Zwolle, the Netherlands; 25 Department of Surgery, Central Manchester University Hospitals NHS Foundation Trust, Manchester, UK; 26 Department of Clinical Genetics and Department of Human Genetics Leiden University Medical Centre, Leiden, The Netherlands; 27 Department of Clinical Genetics, Aarhus University Hospital, Aarhus, Denmark; 28 Department of Biomedicine, Aarhus University, Aarhus, Denmark; 29 Department of Women’s and Children’s health, Division of Obstetrics and Gynecology, Karolinska Institutet, Karolinska University Hospital, Solna S171 76, Stockholm, Sweden; 30 Centre for Epidemiology and Biostatistics, Melbourne School of Population and Global Health, The University of Melbourne, Parkville, VIC Australia; 31 Medical Genetics Unit, University of Florence, Florence, Italy; 32 Center for Hereditary Tumors, HELIOS University Hospital Wuppertal, University Witten/Herdecke, Witten, Germany; 33 Department für Humanmedizin, Universität Witten/Herdecke, Witten, Germany; 34 University of Eastern Finland, Kuopio, Finland; 35 Research Group Inherited Cancer, The Norwegian Radium Hospital, Department of Medical Genetics, Oslo University Hospital, Oslo, Norway

**Keywords:** Lynch syndrome, Hereditary non-polyposis colorectal cancer, Colorectal cancer, Microsatellite instability

## Abstract

**Background:**

We have previously reported a high incidence of colorectal cancer (CRC) in carriers of pathogenic *MLH1* variants *(path_MLH1*) despite follow-up with colonoscopy including polypectomy.

**Methods:**

The cohort included Finnish carriers enrolled in 3-yearly colonoscopy (*n* = 505; 4625 observation years) and carriers from other countries enrolled in colonoscopy 2-yearly or more frequently (*n* = 439; 3299 observation years). We examined whether the longer interval between colonoscopies in Finland could explain the high incidence of CRC and whether disease expression correlated with differences in population CRC incidence.

**Results:**

Cumulative CRC incidences in carriers of *path_MLH1* at 70-years of age were 41% for males and 36% for females in the Finnish series and 58% and 55% in the non-Finnish series, respectively (*p* > 0.05). Mean time from last colonoscopy to CRC was 32.7 months in the Finnish compared to 31.0 months in the non-Finnish (p > 0.05) and was therefore unaffected by the recommended colonoscopy interval. Differences in population incidence of CRC could not explain the lower point estimates for CRC in the Finnish series. Ten-year overall survival after CRC was similar for the Finnish and non-Finnish series (88% and 91%, respectively; p > 0.05).

**Conclusions:**

The hypothesis that the high incidence of CRC in *path_MLH1* carriers was caused by a higher incidence in the Finnish series was not valid. We discuss whether the results were influenced by methodological shortcomings in our study or whether the assumption that a shorter interval between colonoscopies leads to a lower CRC incidence may be wrong. This second possibility is intriguing, because it suggests the dogma that CRC in *path_MLH1* carriers develops from polyps that can be detected at colonoscopy and removed to prevent CRC may be erroneous. In view of the excellent 10-year overall survival in the Finnish and non-Finnish series we remain strong advocates of current surveillance practices for those with LS pending studies that will inform new recommendations on the best surveillance interval.

**Electronic supplementary material:**

The online version of this article (10.1186/s13053-017-0078-5) contains supplementary material, which is available to authorized users.

## Background

Lynch syndrome (LS) is an autosomal dominantly inherited cancer syndrome predisposing to colorectal cancer (CRC) and several extra-colonic malignancies [1]. It is the most common hereditary cause of CRC, accounting for about 3% of the disease. LS is caused by constitutional pathogenic variants of any of four DNA mismatch repair (MMR) genes (*MLH1, MSH2, PMS2* and *MSH6*) or by a deletion in the *EPCAM* gene which leads to *MSH2* inactivation. In mutation carriers, a somatic mutation affecting the second allele leads to defective MMR activity.

Based on the international Prospective Lynch Syndrome Database (PLSD) we have demonstrated that stringent current screening guidelines do not protect fully against the development of colorectal cancer (CRC) in *path_MMR* carriers. This observation is despite undertaking colonoscopies with polypectomies every 3 years or even more frequently [2]. This finding is in contrast to the declared goal of the guidelines [1] that follow-up by colonoscopy and polypectomy aims to prevent CRC – an outcome that we expected to be true when issuing these guidelines. Nonetheless, LS patients undergoing surveillance often survive their first cancer and many develop subsequent cancers, again with a good prognosis, albeit a CRC mortality rate of approximately 10%.

There are many possible reasons why CRC continued to occur in our previously reported series despite follow-up with colonoscopy and polypectomy. These reasons include too great an interval between colonoscopies (Table 1). A high rate of interval CRCs in LS patients having surveillance with screening intervals of over 3 years previously prompted those involved in revising guidelines to recommend shorter surveillance intervals [1, 3–11]. This change was based upon the assumption that a major cause of interval cancers was a fast transition from visible adenomatous polyp to cancer as a consequence of the increased mutation rate associated with MMR deficiency [12]. A 3-year interval between colonoscopies has been shown to reduce the CRC incidence and mortality in LS in a comparative prospective study [3]. However, previous relevant studies [3–7, 13–18] (summarized in Table 2) have provided no definite empirically observed evidence to support what interval between colonoscopies might best prevent CRC in LS, and how protective such an intervention might be.

**Table 1:** National surveillance protocols for colorectal and endometrial cancer

Center	Series cencored	Colonoscopy		Gynecological examination			Reference no. and additional details
		Interval	From-to	Interval	From-to	Modalities in addition to clinical examination	
Finland	2014	3 years	1985–2014	1 year	1995-2014	EB, ultrasound, CA12-5	[3, 4, 8]
Denmark	2014	2 years	1991–2014	2 years	1991-2014	US	[9]
Germany	2014	1 year	1995–2014	1 year	1995-2014	US	[10, 17]
Italy	2013	1-2 years (1 year when age > 40 years; adenoma)	1990–2013	2 years (1 years when age > 35 yrs.)	1990-2013	US, Pap smear	[11]
UK (Manchester)	2014	2 years	1994–2014	1 year	1990-2014	Hysteroscopy, US, CA12-5	[6]
Sweden	2014	2 years	1990–2000	1 year	1992-2014	US, CA 12-5.Some patients: EB	[25, 26]
		18 months	2000–2014				
Australia	2014	1 year	1990–2014	1 year	1990-2005	US, EB, CA12-5	https://www.nhmrc.gov.au/_files_nhmrc/publications/attachments/cp106_0.pdf https://www.nhmrc.gov.au/_files_nhmrc/publications/attachments/cp106_0.pdf
					2005-2014	Risk reducing surgery only	
Spain	2013	1-2 years (1 year when age > 40 years)	1999–2013	1 year	1999-2013	US	Unpublished
The Netherlands	2013	2-3 years	1987–1996	1-2 years	1994-2005	US	[7]
		2 years	1997–2013	1-2 years	2005-2013	US, EB	
UK (Cardiff)	2013	3 years	1991–1994	1 year	1998-2010	US, CA12-5(3 or 4 monthly)	Unpublished Colonoscopy 96% compliance with interval.
		2 years	1994–2013				Gyn: only 27% of eligible women had gyn. Cancer screening. Since 2010 not systematic.
UK (Newcastle)	2014	2 years	1995–2014	No fixed			Unpublished
Norway	2013	3 years	1989–1996	2 years	1989-2013	US, CA12-5	[5]
		2 years (one year when adenoma)	1996–2013				

*US* Transvaginal ultrasound

*EB* Endometrial biopsy

**Table 2:** Studies assessing the effect of colonoscopy surveillance interval in LS

Study	Ref.	Subjects	Inclusion criteria	Setting	CS Interval	Findings
Järvinen, 2000	[3]	252	Amsterdam criteria and LS, gene tested	Prospective, controlled non-randomized trial	3 years	CRC reduced 62% in 15 years compared to not screened.Mortality reduced 65% vs. no CS surveillance. Adherence 93%.
Dove-Edwin, 2005	[13]	290	Amsterdam criteria	Retrospective	3 years	Estimated 72% reduction of CRC mortality when screened. Adherence not reported.
Mecklin, 2007	[4]	420	LS, gene tested	Prospective, observational	3 years	Estimated risk for CRC 22% for women and 35% for men before age 60. No increase in CRC mortality compared to non-carriers. Adherence 98%.
Järvinen, 2009	[14]	242	LS, gene tested	Prospective, controlled non-randomized observational	3 years	CRC incidence 12.4% in 11·5 years, no increase in CRC mortality compared to non-carriers. Adherence 96%.
De vos tot Nederveen Cappel, 2002	[15]	857	Amsterdam criteria or gene tested	Retrospective	2-3 years	10.5% cumulative CRC risk in 10 years under surveillance. Lower tumor stage if CS interval < 2 years.
Stormorken, 2007	[5]	601	Amsterdam criteria or gene tested	Prospective, observational	2-3 years	CRC incidence of LS carriers not increased compared to non-carriers. Adherence not reported.
Newton, 2015	[6]	227	LS, gene tested	Retrospective	2-3 years	CRC incidence 25% at age 70. Adherence 87%.
Stupart, 2009	[16]	178	LS, *MLH1* mutation	Prospective, controlled non-randomized observational	1-2 years	CRC incidence 11% in 5 years compared to 27% if no surveillance. Adherence not reported.
Vasen, 2010	[7]	745	LS, gene tested	Retrospective	1-2 years	CRC cumulative risk 6% in 7·2 years. Adherence not reported.
Engel, 2010	[17]	622	LS, gene tested	Prospective, controlled non-randomized observational	1-2 years	CRC cumulative risk at age 60 23%, early stages. Adherence 81% to 15 months.
Stuckless, 2012	[18]	152	LS, *MSH2* mutation	Retrospective	1-2 years	CRC reduced 71% in 10 years, interval cancers 27% in males and 15% in females. Adherence 44%.

*LS* Lynch syndrome

*CRC* colorectal cancer

*CS* colonoscopy

Among LS patients in our previous reports from PLSD, *path_MLH1* carriers had the highest incidence of CRC despite surveillance colonoscopy, making them the best cohort to examine why CRC continued to occur. The first hypothesis considered was that the large number of carriers reported in PLSD who were from Finland represented a potential confounder. The high overall incidence of CRC observed in *path_MLH1* carriers in PLSD might have arisen because Finland, unlike other countries, had not shortened the recommended interval between colonoscopies from the original 3 year interval advocated many years ago.

Here we report whether or not the high CRC incidence in *path_MLH1* carriers despite colonoscopy surveillance was caused by a high incidence in Finland and investigate time to CRC cancer since last colonoscopy and overall survival.

## Methods

### The Prospective Lynch syndrome database

PLSD contains data stored as an Oracle© relational database. Details on data storage and manipulation have been described in detail earlier [2]. Patients who were subject to prospective follow-up including colonoscopy were reported from LS registries in 13 centers in Europe and Australia. In some cases screening for early detection of endometrial and ovarian cancer were also implemented. Details on the guidelines followed at each contributing center are given in Table 1.

### Study design

The design was a prospective, case-based, open observational study of *path_MLH1* carriers subjected to colonoscopy comparing two groups with different recommended intervals between colonoscopies. The prospective observations recorded included follow-up from first prospectively planned and carried out colonoscopy onwards. Patients with any cancer prior to, or at the age of first colonoscopy (prevalent cancers) were excluded, as were subjects with less than 1 year of prospective observation. The following observations were used: age at first colonoscopy, gender, age at last observation, months from last completed colonoscopy to diagnosis of CRC, age at any cancer together with the ICD diagnosis of the cancer, and age at death.

### Inclusion criteria

All patients included had been subject to prospective follow-up with colonoscopy because of their increased risk for CRC. All were confirmed carriers of *path_MLH1* variants by genetic testing. All *path_MLH1* variants were checked against the LOVD database (http://chromium.lovd.nl/LOVD2/colon_cancer/). Please see our previous report for a more detailed description of inclusion criteria [2].

### Time between colonoscopies in the different centers

All subjects were offered planned regular surveillance colonoscopy and polyp removal according to the guidelines followed at each reporting center (Table 1). The recommended colonoscopy interval in the Finnish national registry protocol was 3 years throughout the study period. From 1997 onwards, all the other centers recommended surveillance intervals of 2 years or less. Thus, only observations from 1997 onwards were included in the present study. Each center kept track of their activities through medical files and/or research registries, and all were able to contribute complete reports for all patients included with no missing values. Whilst these methods do not conform to the stringency of a clinical trial they nonetheless represent the best that can be achieved by a collaborative group of highly interested expert clinicians working in diverse health service systems.

### Surveillance for extra-colonic cancers

Patients in the contributing centers were informed of general cancer awareness but the surveillance for extra-colonic cancers varied across the centers (Table 1).

### Cancer treatment

All cancers were treated according to local standards. Treatment modalities were not considered.

### Events scored

All infiltrating CRCs were scored using the first three positions in the ICD-9 system. Age at each cancer was recorded. Age at death was recorded.

### Annual and cumulative incidence rates

Each patient was observed from age at inclusion to age at last observation or first cancer. For each patient, the number of years observed in each five-year group from 25 years of age onwards was counted. All first cancers were scored according to the age at diagnosis. Annual incidence rates (AIR) for age groups were calculated by dividing the number of cancers observed by the total number of observation years. Each patient was counted once only, irrespective of how many synchronous cancers the patient might have had as first cancers. Later cancers were not considered. Cumulative incidence, denoted by Q, was computed starting at age 25, assuming zero incidence before age 25, using the formula Q(age) = Q(age-1) + [1-Q(age-1)]·AIR(age) where AIR(age) is the annual incidence for the corresponding 5 year interval. The hazard rate H = −ln[1-AIR] was used with standard error estimated as SEH = SEAIR/(1-AIR). The standard error, denoted by SEQ, was computed in two steps and 95% confidence intervals were estimated. Follow-up continued after the occurrence of first cancers, and all patients were either reported to be alive or validated to be alive in a population register when censored. See our previous report [2] for a more detailed description of these methods. Differences in time-to-events were also calculated by the Kaplan-Meier algorithm and Mantel-Cox *p*-values when appropriate.

### Survival

Crude survival was calculated by the Kaplan-Meier algorithm and Mantel-Cox p-values as time from first cancer to last observation/death. Cancer stages at diagnosis and causes of death were not considered in this report.

## Results

There were 7924 observation years in the final analysis from 430 males and 514 females (*n* = 944). The Finnish series included 4625 and the non-Finnish series 3299 observation years after first colonoscopies. Ages at inclusion were similar for both series (35.2 years and 36.1 years, respectively; *p* > 0.05). Baseline characteristics of the study population and the classification of *path_MLH1* variants are presented in Table 3.

**Table 3:** Baseline characteristics of the study population

		All subjects	3-year interval (Finnish)	1-2-year interval (non-Finnish)
Observation years		7928	4625	3299
Number of subjects		944	505	439
Gender	Male	430	246 (48.7%)	184 (41.9%)
	Female	514	259 (51.3%)	255 (58.1%)
Age at inclusion	(Mean, SD)	35.5 (11.7)	35.2 (12.1)	36.1 (11.0)
Follow-up time	(Mean, SD)	8.4 (5.7)	9.2 (5.9)	7.5 (5.2)

*SD* standard deviation

### Classification of *path_MLH1* variants

Seven hundred and six (75%) of the patients carried *path_MLH1* variants found in LOVD, and 238 of variants (25%) were not found in LOVD. Among the 706 found in LOVD, 691 (98%) were pathogenic (class 5) and 15 (2%) were probably pathogenic (class 4). Among the 505 in the Finnish series 406 (80%) and four (0.7%) had class 5 and class 4 variants, Among the non-Finnish series 285 (65%) had class 5 and 11 (2.5%) class 4 variants in LOVD, Among the 238 variants not reported to LOVD 95 were detected in the Finnish series and 143 in the non-Finnish series. All of them were eventually classified as class 5 and 4 based on the combined assessment of two of the co-authors (IF, JS) following the updated InSight rules [19].

### Cumulative incidences of cancers

**Fig. 1:**
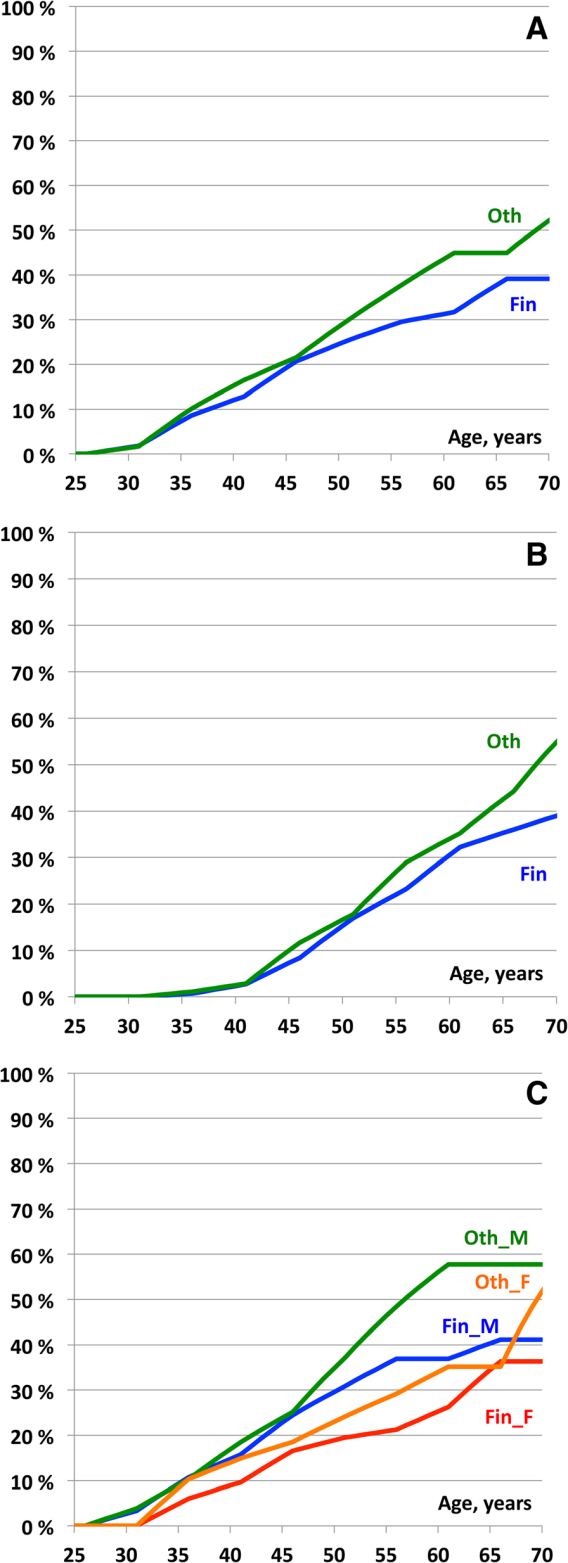
**a** Calculated cumulative incidence for colorectal cancer in the 3-year and non-Finnish series. Fin = Finnish series (blue line). Oth = non-Finnish series (green line). **b** Calculated cumulative incidence for extra-colonic cancer in the 3-year and non-Finnish series. Fin = Finnish series (blue line). Oth = non-Finnish series (green line). **c** Calculated cumulative incidence for colorectal cancer by gender in the 3-year and non-Finnish series. Fin_M = Finnish series, males (blue line). Fin_F = Finnish series, females (red line). Oth_M = non-Finnish series, males (green line). Oth_F = non-Finnish series, females (orange line)

A total of 101 CRCs and 83 extra-colonic cancers were diagnosed during the follow-up period. Despite less frequent colonoscopy, the cumulative incidences of CRC were modestly lower for the Finnish series compared to the non-Finnish series in all age groups (39.2%; 95% confidence interval 29.4–48.9 vs 53%; 39.6–68) but not significantly so. Regarding extra-colonic cancers, similar observations were made (39.7%; 28.2–51.2 vs 57.2%; 41–73.5, respectively). The cumulative incidence rates categorized by 10-year intervals from age 25 are shown in Table 4 and Fig. 1a and b. Additional file 1: Table S1 presents the annual incidence rates of cancers categorized by five-year intervals from 25 to 70 years.

**Table 4:** Cumulative incidences from 25 years of age and 95% confidence intervals for colorectal cancer and extra-colonic cancer

		3-year interval (Finnish)				1-2-year interval (non-Finnish)			
Current age	Age stop	Obs years	#Ca	Cumulative incidence (%)	95% CI	Obs years	#Ca	Cumulative incidence (%)	95% CI
Colorectal cancer									
All									
25	40	1982	19	12.9	[7.4–18.3]	1299	17	16.4	[9.2–23.7]
25	50	3301	40	25.4	[18.6–32.3]	2424	36	30.0	[21.7–38.3]
25	60	4070	47	31.7	[24.0–39.4]	2935	48	44.9	[34.6–55.2]
25	70	4330	51	39.2	[29.4–48.9]	3094	50	53.8	[39.6–68.0]
Male									
25	40	1026	12	15.7	[7.5–23.9]	610	9	18.5	[7.2–29.7]
25	50	1682	25	30.8	[20.7–40.9]	1110	21	37.0	[24.3–49.8]
25	60	2075	29	36.9	[26.1–47.7]	1288	28	57.8	[41.5–74.0]
25	70	2189	30	41.1	[28.2–54.0]	1365	28	57.8	[41.5–74.0]
Female									
25	40	956	7	9.6	[2.8–16.4]	689	8	14.8	[5.3–24.4]
25	50	1623	15	19.4	[10.6–28.3]	1314	15	24.1	[13.4–34.8]
25	60	2010	18	26.2	[15.2–37.2]	1646	20	35.2	[22.0–48.3]
25	70	2156	21	36.4	[22.1–50.6]	1729	22	55.3	[30.5–80.0]
Extra-colonic cancer									
All									
25	40	1982	4	2.7	[0.1–5.3]	1299	3	2.9	[0.0–6.1]
25	50	3301	24	16.9	[10.7–23.2]	2424	22	17.8	[11.0–24.6]
25	60	4070	39	32.2	[23.5–40.9]	2934	35	35.3	[24.8–45.7]
25	70	4330	42	39.7	[28.2–51.2]	3094	41	57.2	[41.0–73.5]

*Obs years* observation years at age group

*#Ca* number of cancers during observation

*CI* confidence interval

The similar age of inclusion allowed us to make comparisons of time from inclusion to diagnosis of CRC using the Kaplan-Meier algorithm. In the Finnish series, 3 and 11% of carriers were diagnosed with CRC after 5 and 10 years of follow-up, respectively, compared to 5 and 14%, respectively in the non-Finnish series (*p* > 0.05). The differences were modestly larger in males than in females (Fig. 1c). The percentages of carriers developing extra-colonic cancers were also similar: 3 and 9% at 5 and 10 years after inclusion in the Finnish series compared to 3 and 11% in the non-Finnish series (p > 0.05). In sum, none of the differences were significant and all point estimates were similar or lower in the Finnish series.

### Correlation with population incidence of CRC

Next, we explored the correlation between observed CRC incidences in *path_MLH1* LS patients and the corresponding population incidences of CRC. The relative incidence of CRC in the Finnish population compared to the other countries was 0.80 [the age-standardized incidence rate in Finland was 35.0 per 100,000 versus a mean of 43.6 per 100,000 for the other reporting countries (http://eco.iarc.fr/EUCAN/; downloaded October 2015)]. By comparison, the observed ratio of cumulative risk for CRC at 70 years in the Finnish series of *path_MLH1* carriers compared to the others was similar: 0.72 (39.2% in the Finnish series versus the average of 54.8% in other countries combined).

### Time since last colonoscopy to CRC and survival

The mean time from last colonoscopy to CRC did not differ between the Finnish and non-Finnish series irrespective of the interval between colonoscopies. It was 32.7 (SD 13.6) months for the Finnish series and 31.0 (SD 23.4) months for the non-Finnish series (p > 0.05). Times since last colonoscopy to CRC in the two series by 6 months periods are shown in Table 5. Of note, the rates of interval cancers occurring before the next planned 2-yearly or 3-yearly colonoscopy were similar in both series at 56.9% and 50% in the Finnish and the others respectively (Table 5).

**Table 5:** Months since last colonoscopy with no cancer to colorectal cancer diagnosed

	3-year interval (Finnish)			1-2-year interval (non-Finnish)		
Months since last colonoscopy	Number CRC	Cumulative number CRC	Cumulative %	Number CRC	Cumulative number CRC	Cumulative %
<6	0	0	0%	0	0	0%
7–11	2	2	3.9%	5	5	10%
12–17	3	5	9.8%	13	18	36%
18–23	4	9	17.6%	7	25	50%
24–29	17	26	51%	6	31	62%
30–35	3	29	56.9%	4	35	70%
36–41	14	43	84.3%	3	37	74%
42–47	3	46	90.2%	3	40	80%
48–120	5	51	100%	10	50	100%

*CRC* colorectal cancer

Moreover, there were no differences in the survival of CRC in *MLH1* carriers in the two series. Five- and ten-year overall survival after CRC was 90% (95% confidence interval: 78-96%) and 88% (75-95%), respectively, in the Finnish series compared to 96% (85-99%) and 91% (78-97%) in the 1-2-year interval series (p > 0.05). There were not enough deaths for meaningful stratification by time since last colonoscopy with respect to survival. Finally, there were no differences in survival of extracolonic cancers. Five- and ten-year survival after extra-colonic cancer was 79% (64-89%) and 79% (64-89%), respectively, in the Finnish series compared to 82% (68-92%) and 82% (69-92%) in the 1-2-year interval series (p > 0.05).

## Discussion

This study showed that the high incidence of CRC observed in our combined international series of *path_MLH1* carriers was not caused by a higher incidence in the Finnish series compared to the others. In contrast to what we expected, the CRC incidence in the Finnish series was lower than in the others, but not significantly so.

First, we considered the possibility that the trend towards a lower CRC incidence in the Finnish series could reflect the lower population incidence of CRC in Finland. In fact, the observed ratio of cumulative risk for CRC at 70 years showed a similar pattern in the Finnish and non-Finnish series. Disease expression differences between Finnish and non-Finnish *path_MLH1* carriers could reflect population-specific environmental and behavioural factors or a lower penetrance of the Finnish founder *path_MLH1* variants compared to other *path_MLH1* variants but this study shows that these factors cannot have a major impact on the observed high cumulative risk of CRC in *path_MLH1* carriers in the total PLSD cohort of *path_MLH1* patients.

Secondly, we examined whether or not the Finnish patients were actually colonoscoped less frequently than the others. We found that the time between last colonoscopy and CRC was similar for both series irrespective of the recommendations in place. Also, it was evident that a proportion of CRCs in both series could be classified as interval cancers (Table 5). In particular, the rates of interval cancers that occurred before the planned next 2-yearly or 3-yearly colonoscopy were 56.9% and 50% in the Finnish series and the others, respectively (Table 5). While the organizational aspects of surveillance at the non-Finnish centers were heterogenous and details that might impact our observations were not readily available, the organization of surveillance in the Finnish series displays two key features that are critical for the interpretation of our findings: there are special and dedicated booking and call back systems in place to follow all identified LS carriers and the reported adherence to colonoscopy intervals has been consistently very high throughout the years [4, 8]. Thus, in the Finnish series non-compliance/adherence with local surveillance guidelines cannot explain the occurrence of all cancers and the pattern of time from last colonoscopy to CRC suggests that only a limited proportion of CRCs would have been prevented by reducing the interval from 3 to 2 years.

Our study has a number of strengths including: (i) a large number of observation years; (ii) a focus on *path_MLH1* carriers showing a high cumulative risk thereby avoiding mixing of carriers of *path_MMR* variants in different MMR genes and (iii) the robustness of the dataset collated based upon prospectively observed outcomes. On the other hand, several limitations need to be highlighted: (i) in spite of the number of observation years included our sample size is still limited. Stochastic variation cannot be completely ruled out and our observations do not exclude a significant impact of differences in population prevalence of CRC on LS expressivity; and (ii) only a detailed analysis of the interval, quality and reported findings of the colonoscopies will allow a refined assessment of the impact of this confounder. However, such an analysis will not change the critical observation that time between colonoscopy and CRC is essentially the same in both series.

The prospective health outcome observations presented here challenge the dogma that the performance of very frequent colonoscopies, many of them performed in expert centers, has a strong primary preventive effect on CRC incidence in LS. It is notable that despite surveillance, the expert centers with good recall systems in place that have contributed their data to this study have not succeeded in preventing CRC as has been achieved in non-LS familial CRC [20]. Consequently, our observations set the foundations for a more detailed analysis of the determinants of this lack of success. In this regard, there is biological evidence that CRC in LS may arise directly without a precursor polyp although the relative contribution of this distinct natural history to the totality of CRCs observed is a matter of controversy [21–24]. Thus, it is possible that even colonoscopies achieving the best quality standards would not be able to prevent all CRCs in LS.

Whether shorter colonoscopy interval could result in lower CRC stage needs to be addressed in future studies with cancer stage included. We are in the process of expanding our database in this regard.

## Conclusions

In summary, we tested whether or not the high incidence of CRC in *path_MLH1* carriers observed in our previous reports was caused by a distinct high incidence in the Finnish series in which 3-yearly colonoscopy was recommended. In the present report we show that the cumulative risk of CRC is high for *path_MLH1* carriers undergoing colonoscopic surveillance irrespective of the specific characteristics of their country of origin and the associated factors that may have an impact on the expression of the disease. The contribution of more observation years from countries with different population CRC incidences will be needed to formally test the hypothesis that population CRC incidence correlates with LS expression. The lack of a difference in the time between last colonoscopy and CRC in the two series investigated in this study challenges current beliefs regarding colonoscopy intervals in LS surveillance. Our findings mandate a detailed study that will eventually inform policy makers. Finally, in view of the excellent 10-year overall survival in the Finnish and non-Finnish series we remain strong advocates of current surveillance practices for those with LS pending studies that will inform new recommendations on the best surveillance interval.

## Additional file

Additional file 1: Table S1. Annual incidence rates (AIR) and 95% confidence intervals for colorectal cancer and extra-colonic cancer. (DOCX 15 kb)

## Abbreviations

AIR: Annual incidence rate
CI: Confidence interval
CRC: Colorectal cancer
ICD9: International Classification of Disease, 1975 revision.
InSiGHT: International Society for Gastrointestinal Hereditary Tumours
LOVD: Leiden Open Variation Database
LS: Lynch Syndrome
MMR: Mismatch repair
*path_MLH1*: Pathogenic (disease-causing) variant of the *MLH1* gene
*path_MSH2*: Pathogenic (disease-causing) variant of the *MSH2* gene
*path_MSH6*: Pathogenic (disease-causing) variant of the *MSH6* gene
*path_PMS2*: Pathogenic (disease-causing) variant of the *PMS2* gene
