# Supplementary material for: Colorectal cancer incidence in path_MLH1 carriers subjected to different follow-up protocols: a Prospective Lynch Syndrome Database report
Source: Hered Cancer Clin Pract. 2017 Oct 10;15:18. doi: 10.1186/s13053-017-0078-5 (PMC5635542; doi:10.1186/s13053-017-0078-5)
Supplement: Additional file 1: Table S1. — Annual incidence rates (AIR) and 95% confidence intervals for colorectal cancer and extra-colonic cancer. (DOCX 15 kb) [file 13053_2017_78_MOESM1_ESM.docx]

Table S1. Annual incidence rates (AIR) and 95% confidence intervals for colorectal cancer and extra-colonic cancer

| Colorectal cancer | | | | | | | | | |
| --- | --- | --- | --- | --- | --- | --- | --- | --- | --- |
| 3-year interval (Finnish) | | | | |  | 1-2-year interval (non-Finnish) | | | |
| Age | Obs | #Ca | AIR | 95% CI |  | Obs | #Ca | AIR | 95% CI |
| 25-30 | 539 | 2 | 0.0037 | 0.0004-0.0134 |  | 296 | 1 | 0.0034 | 0.0000-0.0189 |
| 30-35 | 708 | 10 | 0.0141 | 0.0068-0.0260 |  | 450 | 8 | 0.0178 | 0.0078-0.0351 |
| 35-40 | 735 | 7 | 0.0095 | 0.0038-0.0196 |  | 553 | 8 | 0.0145 | 0.0063-0.0286 |
| 40-45 | 750 | 14 | 0.0187 | 0.0103-0.0313 |  | 637 | 8 | 0.0126 | 0.0055-0.0248 |
| 45-50 | 569 | 7 | 0.0123 | 0.0049-0.0253 |  | 488 | 11 | 0.0225 | 0.0112-0.0403 |
| 50-55 | 442 | 5 | 0.0113 | 0.0036-0.0220 |  | 345 | 8 | 0.0231 | 0.0101-0.0457 |
| 55-60 | 327 | 2 | 0.0061 | 0.0063-0.0583 |  | 165 | 4 | 0.0242 | 0.0067-0.0618 |
| 60-65 | 175 | 4 | 0.0229 | 0.0063-0.0583 |  | 102 | 0 | 0.0000 | 0.0000-0.0363 |
| 65-70 | 85 | 0 | 0.0000 | 0.0000-0.0435 |  | 58 | 2 | 0.0345 | 0.0034-0.1241 |
| Total | 4330 | 51 | 0.0118 | 0.0088-0.0155 |  | 3094 | 50 | 0.0162 | 0.0120-0.0213 |
|  | | | | | | | | | |
| Extra-colonic cancer | | | | | | | | | |
| 3-year interval (Finnish) | | | | |  | 1-2-year interval (non-Finnish) | | | |
| Age | Obs | #Ca | AIR |  |  | Obs | #Ca | AIR | 95% CI |
| 25-30 | 539 | 0 | 0.000 | 0.0000-0.0069 |  | 296 | 0 | 0.0000 | 0.0000-0.0125 |
| 30-35 | 708 | 1 | 0.0014 | 0.0000-0.0079 |  | 450 | 1 | 0.0022 | 0.0000-0.0124 |
| 35-40 | 735 | 3 | 0.0041 | 0.0008-0.0120 |  | 553 | 2 | 0.0036 | 0.0004-0.0130 |
| 40-45 | 750 | 9 | 0.0120 | 0.0055-0.0228 |  | 637 | 12 | 0.0188 | 0.0097-0.0330 |
| 45-50 | 569 | 11 | 0.0193 | 0.0097-0.0346 |  | 488 | 7 | 0.0143 | 0.0057-0.0294 |
| 50-55 | 442 | 7 | 0.0158 | 0.0063-0.0326 |  | 345 | 10 | 0.0289 | 0.0139-0.0532 |
| 55-60 | 327 | 8 | 0.0245 | 0.0107-0.0483 |  | 165 | 3 | 0.0182 | 0.0036-0.0533 |
| 60-65 | 175 | 2 | 0.0114 | 0.0011-0.0411 |  | 102 | 3 | 0.0294 | 0.0059-0.0863 |
| 65-70 | 85 | 1 | 0.0118 | 0.0000-0.0659 |  | 58 | 3 | 0.0517 | 0.0103-0.1517 |
| Total | 4330 | 42 | 0.0097 | 0.0070-0.0131 |  | 3094 | 41 | 0.0133 | 0.0095-0.0180 |

Obs = observation years at age group.

#Ca = number of cancers during observation.

AIR = annual incidence rate.

CI = confidence interval.
